# Rapid estimation of viral emission source location via genetic algorithm

**DOI:** 10.1007/s00466-021-02138-7

**Published:** 2022-01-25

**Authors:** L. M. Clemon

**Affiliations:** 1 University of Technology Sydney, Ultimo, Australia; 2 Present Address: Ultimo, NSW Australia

**Keywords:** Stochastic, Concentration, Particles, Genetic algorithm, Virus

## Abstract

Indoor spread of infectious diseases is well-studied as a common transmission route. For highly infectious diseases, like Sars-CoV-2, considering poorly or semi ventilated areas outdoors is increasingly important. This is important in communities with high proportions of infected people, highly infectious variants, or where spread is difficult to manage. This work develops a simulation framework based on probabilistic distributions of viral particles, decay, and infection. The methodology reduces the computational cost of generating rapid estimations of a wide variety of scenarios compared to other simulation methods with high computational cost and more fidelity. Outdoor predictions are provided in example applications for a gathering of five people with oscillating wind and a public speaking event. The results indicate that infection is sensitive to population density and outdoor transmission is plausible and likely locations of a virtual super-spreader are identified. Outdoor gatherings should consider precautions to reduce infection spread.

## Introduction

The recent pandemic has spurred broader interest in particulate based transmission of diseases. A recent review of spread in Wuhan reveals a substantially higher transmission rate than severe acute respiratory syndrome (SARS) and Middle East respiratory syndrome (MERS) [1]. Transmission studies have focused primarily on indoor settings where transmission rates are known to be significant [2–4]. Indoor settings represent a significant and known transmission vector for airborne spread and are thus the focus of many public health researchers [5–8]. However, community spread is difficult to trace in many countries, particularly with more infectious strains ([9, 10]) and points to outdoor infection as an additional possible vector [11]. Such spreading would be expected to be more prevalent in dense populations with poor adherence to infection control measures and are more difficult to contact trace. A recent study provides a simulation framework for macro transmission by human-human tracking [12], as well as the role of atmospheric conditions on transmission rates [13]. Previous localized simulations have focused on particle methods using computationally intensive methods like discrete element models (DEM) or computational fluid dynamics (CFD), such studies include for a single indoor cough [14, 15] and computational fluid-particle dynamics showing dispersion by bulk fluid transport plays a significant role [16] . The computing infrastructure required for these studies scales rapidly with the number of particles and size of the simulation space. Such simulations at scales relevant in particle number and geometric length for outdoor environments require significant computing power and parallelization [17–19]. In contrast, to this computationally intensive simulation practice, quasi-analytical and statistical averaging approaches can be employed with less intensive numerical simulation for rapid estimation. This computationally simplified approach can give rapid approximate reconstruction of transmission events and insight as to where to focus more detailed study, when necessary.

An adaptable framework and numerical model for the airborne infection spread is presented. A base model is developed for the generation and transport of infectious or hazardous particles in the environment. This base model is incorporated into a genetic algorithm to search for likely source locations which aids in recreating transmission events. This framework provides an aid in further developing health guidelines as communities transition from avoiding social interactions to living with altered social interactions and in reverse engineering spreading events. Rapid simulation is tuned using epidemiological and infections disease data for prediction of community based infection transmission of virus containing aerosols. In addition, more granular resolution simulations that provide parameter values are incorporated, such as the distribution of a cough [14], the size of emitted particles [20], or the half-life of viral particles [21, 22]. This model is applied in outdoor settings where public health has minimally evaluated thus far. An outdoor event is simulated and presented as an example, identifying the likely location of a super-spreader using a genetic algorithm. Spreader localisation is possible and rapid in this framework due to the low computational cost of the particle dispersion model.

## Methodology

The framework is split into three models that interact to capture system dynamics: (a) source model, (b) particulate model, and (c) environment model. These three models interact as the simulation progresses forward in time. In the application context of an infectious disease models (a) and (b) represent people and the viral particles, respectively. Several researchers have evaluated people and their propensity to shed viruses with or without symptoms [5, 23, 24]. Shedding is incorporated into the person (source) model and captures the needed quantity of particulate viral matter ejected into the environment. Thus, the prevalence of symptoms is not explicitly considered nor needed in applying this framework to infectious diseases and it can be used to simulate asymptomatic spreading events.

Much research is focused on evaluating the virus properties or macro transmission dynamics [1, 2, 21]. This research is synthesized into a distribution of virus particle size and the life of viral particles in the environment. A half-life model is applied for viral particles computed from data collected by [21]. While detailed particulate simulations are useful in modelling particulate flow ([25–29]), a binned distributional weighting model is used to reduce computational requirements. This greatly reduces the computational time and memory while capturing a profile of virus spread in the local area. The environment model allows for inclusion of ambient and oscillating wind conditions for the selected scenarios. Two examples are presented: one demonstrating a simple arrangement and oscillating wind condition, and an example analysis inspired by a well-documented outdoor speech.

The change in concentration over time is modeled as a diffusion advection equation with a dispersion term for airflow induced spread and a source/sink term for particle generation by infected people. The final concentration is accumulated over time in an forward-time step initial value problem setup.

1 $$\begin{aligned} \frac{d u}{d t}= & {} \underbrace{\nabla \cdot (D\nabla u)}_{diffusion} - \underbrace{\nabla \cdot (\varvec{\nu } u)}_{dispersion}+ \underbrace{R - K}_{sources/sinks} \end{aligned}$$

2 $$\begin{aligned} \frac{du}{dt}= & {} \left( {\delta }u_{d} + {\delta }u_{s} + R - K\right) \end{aligned}$$

where *u* is the concentration of viral particles, *t* is time, *D* is the diffusion rate, $\varvec{\nu }$ is the particle velocity, *R* is a particle generation term, and *K* is a particle absorption term. This model contains three main components that are handled individually, then combined to advance the simulation in time as:

3 $$\begin{aligned} u_{t+1} = u_{t} + \frac{du}{dt} \varDelta t \end{aligned}$$

### Source model

A source (person) model is defined that establishes a set of individual characteristics for each person, or agent, in the environment. For infectious disease spread, this incorporates the breathing rate, presence of infection, viral shedding, location, and emitted particle size distribution. These characteristics define how the person interacts with the environment. The individualized parameters allow for the rapid simulation of the unique people in spreading events observed in practice. Super-emitters are a key type of person to consider ([23, 30, 31]) and can be included by changing just one parameter: shedding rate. Each person is given a unique breathing rate that captures the breathing or talking rate, which is known to influence particle generation [32, 33]. In addition, the effects of increased breathing rate induced by exercise are included through a single parameter change.

For infected and contagious people, the virus shedding rate is multiplied by the unique breathing rate to indicate the quantity of viral particles emitted into the environment. The concentration of infections particles added in the grid space(s) occupied by an emitting person (source) are computed by dividing the total number of emitted particles in a time step by the occupied area:

4 $$\begin{aligned} R = \frac{\gamma {\dot{b}} s }{A } \varDelta t \end{aligned}$$

where $\gamma$ is the density of particles per volume of emitted aspirate, ${\dot{b}}$ is the number of breaths per minute, *s* is the aspirate, or water volume, emitted per breath, *A* is the affected area and $\varDelta t$ is the time step size in minutes. This allows for adjustment of the spatial discretization without compromising the spatial distribution of particle emissions. The distribution of aspirate particle sizes is modelled as set of discrete binned layers.

5 $$\begin{aligned} {\varvec{{d}}}= [d_{p,l} ]_{l=1}^{m} \end{aligned}$$

where $d_{p}$ is a particle diameter, ${\varvec{{d}}}$ is the vector of *m* binned particle sizes (number of layers), and *l* is the layer index. Particle sizes are distributed into layers to account for the changing transport and generation/decay rates of differing sizes. Each layer is handled separately in the environment model.

For non-infected people, absorption of infectious particles is modelled by an absorption coefficient on the breathing rate, multiplied by the concentration in the grid space occupied by the person, and summed across all particle sizes. The absorption of infectious particles is summed across all particle size layers in the area occupied by the person:

6 $$\begin{aligned} L_{t,h} = L_{t-1,h} + \sum _{l} a {\dot{b}} u_{t-1,l}(i_{h},j_{h}) \end{aligned}$$

where *L* is the total viral load in a person, *a* is the individual absorption coefficient, *h* is an index for each person, and *l* is an index for each particle size layer. If a person’s total viral load exceeds their individual infection threshold, they are deemed infected. This allows inclusion of long-duration exposure accumulation.

### Virus model

People labelled contagious are modelled as shedding viral particles. Viral particles are modelled as a constant per volume of aspirate. The volume of aspirate emitted by a person is based on a person’s shedding rate. The higher the shedding rate, the more aspirate and thus, more viral particles. This couples virus particle generation rate to the breathing rate of each person in the person model. Virus quantity is constant per volume in droplets (larger droplets contain a larger virus quantity). A distribution of droplet sizes is defined as several binned sizes. The distribution of particle sizes is divided into 4 size range bins for the case studies below, but could be adjusted as needed. These ranges have different aerodynamic properties. For each range, a separate particle size layer, *l*, is created to track spread for that particle bin. The cumulative viral load in a voxel is tallied to compute the total viral concentration in that voxel. Viral particles decay over time in the environment due to evaporation, sterilisation processes like ultraviolet light exposure, and adhesion to surfaces (removing them from being inhaled). This viral decay is modeled as an exponential decay with a decay rate, *r*, which can be varied by particle size layer, *l*. A constant numerical value is computed using experimental data from Van Doremalen [21] for the example results in Sects. 3.1, 3.2 .

7 $$\begin{aligned} K = u_{t-1,l}e^{-r_{l}\varDelta t} \end{aligned}$$

### Environment model

Particulate spread is considered in two modes: (1) diffusion and (2) wind-induced dispersion. Diffusion is modelled as:

8 $$\begin{aligned} {\delta }u_{d} = \nabla \cdot (D\nabla u) \end{aligned}$$

where *u* is the virus concentration and *D* is diffusion coefficient. Diffusion is discretized into a centered-step finite difference formulation:

9 $$\begin{aligned} \alpha= & {} \frac{D}{\triangle x^{2}}; \text { } \beta = \frac{D}{\triangle y^{2}} \end{aligned}$$

10 $$\begin{aligned} {\delta }u_{d}= & {} \alpha (u_{i-1,j,l} + u_{i+1,j,l}) - u_{i,j,l}(2\alpha + 2\beta ) \nonumber \\&+ \beta (u_{i,j-1,l} + u_{i,j+1,l}) \end{aligned}$$

where $\beta$ and $\alpha$ are intermediate calculation parameters, *x* and *y* are coordinate locations, and *i*, *j*, *l* are indices for location and layer.

Diffusion can be modelled from the Stokes-Einstein diffusion model following the recognized applicability of this method for human breadth [29] and aids in prioritizing computational simplicity for the overall model. For this application, the Cunningham slip correction factor, $C_{S}$, is needed and applied based on particle size. This gives our diffusion coefficient for each bin of the particle distribution:

11 $$\begin{aligned} D_{l} = \frac{k_{Boltz}T_{abs}C_{S}}{3 \pi \mu d_{p,l}} \end{aligned}$$

where $T_{abs}$ is the absolute temperature and $\mu$ is the fluid viscosity.

Following diffusion, a wind dispersion effect is calculated. Dispersion is the spreading of particles, primarily due to drag forces induced by wind. We define this dispersion as:

12 $$\begin{aligned} {\delta }u_{s} = \nabla \cdot (\varvec{\nu } u) \end{aligned}$$

where $\varvec{\nu }$ is the effective particle velocity, and

13 $$\begin{aligned} \varvec{\nu }={\varvec{{v}}}- {\varvec{{v}}}_{terminal} \end{aligned}$$

where ${\varvec{{v}}}$ is the wind velocity. A vector field of wind conditions is updated at each time step. Any known wind condition can be used. More computationally intensive and granular models use a Langevin formulation for aerosol particle transport. This is quite intensive for tracking a large population of particles. We reduce the cost of this by pre-computing a solution for each bin of particle size at terminal velocity using Stokes flow.

We define the frictional drag force as:

14 $$\begin{aligned} f_{f} = 3\pi \mu d_{p}/C_{S} \end{aligned}$$

where $C_{S}$ is the Cunningham correction factor (see Table 1 for values), $\mu$ is the dynamic viscosity, and $d_{p}$ is the particle diameter. The maximum velocity of the particle travelling with the wind is the wind velocity minus the terminal velocity and is reached when the acceleration is zero.

**Table 1:** Binned particle properties

$d_{p}$	$C_{S}$	*D*
$\mu m$		$m^2/s$
0.2	1.9	2.14e-10 [29]
0.5	1.3	6.08e-11 [29]
1	1.2	2.66e-12 [29]
2	1.1	1.16e-12
5	1	4.63e-13
10	1	2.31e-13

The maximum velocity of these spherical particles is bounded by the fluid (wind) velocity, ${\varvec{{v}}}$, and the physical properties of the aspirate. We compute the maximum particle velocity as the fluid velocity less the terminal velocity. In this case the terminal velocity is used to determine the maximum drag on the particle.

15 $$\begin{aligned} \varvec{\nu } = {\varvec{{v}}}- \frac{\rho _{p} d_{p}^2gC_{S}}{18 \mu } \end{aligned}$$

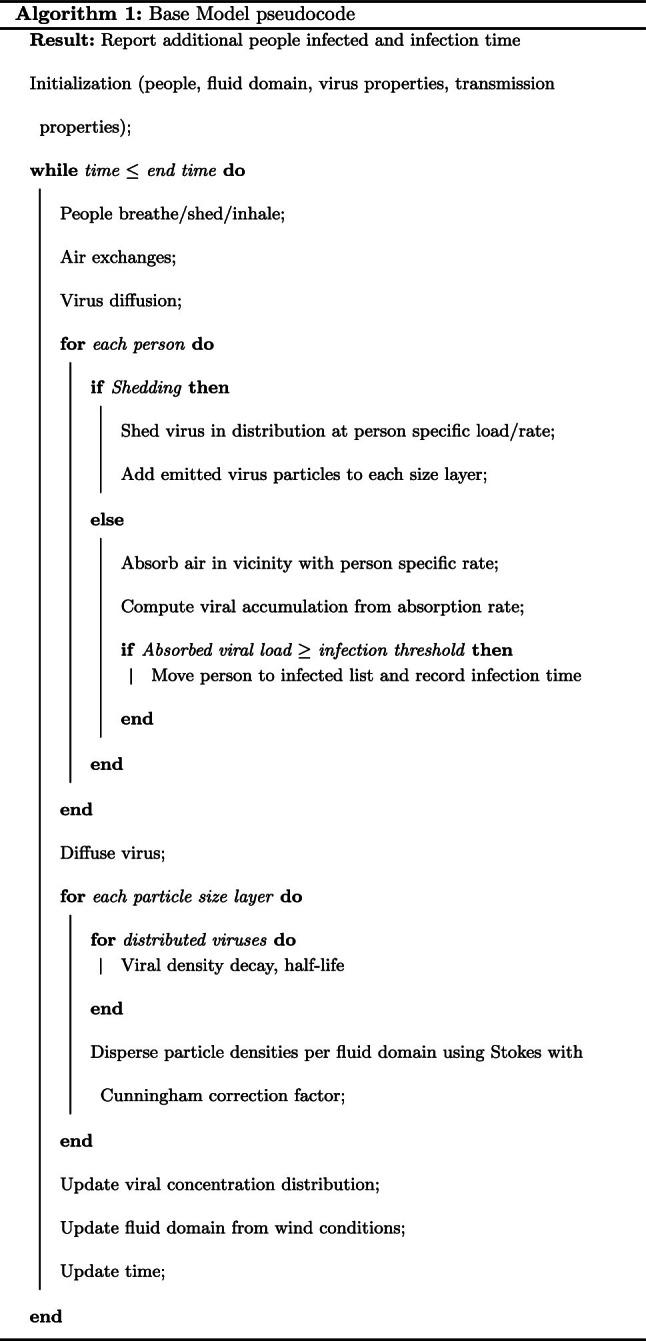


Here we simplify this individual particle tracking to an average based on a pre-computed solution for each selected particle size in the binned size distribution.

This dispersion requires identifying the wind direction relative to each voxel. An upwind spatial stepping method is required for numerical stability ([34, 35]):

16 $$\begin{aligned} {\varvec{{n}}}= & {} \frac{{\varvec{{v}}}}{||{\varvec{{v}}}||} = \frac{v_{x}{\hat{i}} + v_{y}{\hat{j}}}{||{\varvec{{v}}}||} = n_{x}{\hat{i}} + n_{y}{\hat{j}} \end{aligned}$$

17 $$\begin{aligned} \nu _{x}u= & {} {\left\{ \begin{array}{ll} \nu _{x}(u_{i,j} - u_{i-1,j}) &{} n_{x} > 0 \\ \nu _{x}(u_{i+1,j} - u_{i,j}) &{} n_{x} < 0 \end{array}\right. } \end{aligned}$$

18 $$\begin{aligned} \nu _{y}u= & {} {\left\{ \begin{array}{ll} \nu _{y}(u_{i,j} - u_{i,j-1}) &{} n_{y} > 0 \\ \nu _{y}(u_{i,j+1} - u_{i,j}) &{} n_{y} < 0 \end{array}\right. } \end{aligned}$$

where ${\varvec{{n}}}$ is the unit vector indicating the wind direction for grid location (*i*, *j*)

Stability criteria for simulation considers the criteria for forward-time center-spaced finite difference, and upwind method. For the upwind forward-time the Courant-Friedrichs-Levy stability criteria is needed [35, 36]. The condition indicates that the wave velocity must be slower than the numerical spread rate of the scheme. Thus, for a given grid size and wind speed the minimum requisite time-step is computed and used globally:

19 $$\begin{aligned} \triangle t \le \min _{l}\left( \frac{\triangle x^{2}}{2D},\frac{\triangle y^{2}}{2D},\frac{\triangle x}{v_{max}}, \frac{\triangle y}{v_{max}}\right) \end{aligned}$$

### Genetic algorithm

A genetic algorithm is used to search the simulation space to identify the likely viral emission source and location in a known spreading event. This is constructed by fixing the known parameters of the event and searching over ranges of uncertain variables. A recent example of an outdoor speech is evaluated to demonstrate the process. This example is inspired by a known event in Washington, D.C. where 7 individuals were later reported to become infected with COVID-19 [37]. The genetic algorithm starts by generating a sample string where entries are sampled randomly from the available ranges of uncertain variables, for example:

20 $$\begin{aligned} \varLambda _{z} = \lbrace {\varvec{{d}}},\mu ,{\dot{b}},h(n,x,y),{\varvec{{v}}}(A_{x},B_{x},A_{y},B_{y},\omega _{x},\omega _{y}) \rbrace \end{aligned}$$

The sample string, $\varLambda _{z}$ is used as an input into the base model in Algorithm 1 for the specified event duration and fixed parameters. The results of evaluating string $\varLambda _{z}$ must then be evaluated with a scoring criteria. This scoring criteria must be defined for the specific scenario of interest. In the case of identifying the most likely location for a super-spreader with known infection cases, this criteria takes the form of a global maximum exposure of all known cases:

21 $$\begin{aligned} {\varvec{L}}= & {} \lbrace L_{h} \rbrace _{h=1}^{N} = {\mathcal {F}}(\varLambda _{z}) \end{aligned}$$

22 $$\begin{aligned} S_{z}= & {} \sum _{h}L_{h} - \lambda \sigma ({\varvec{L}}) \end{aligned}$$

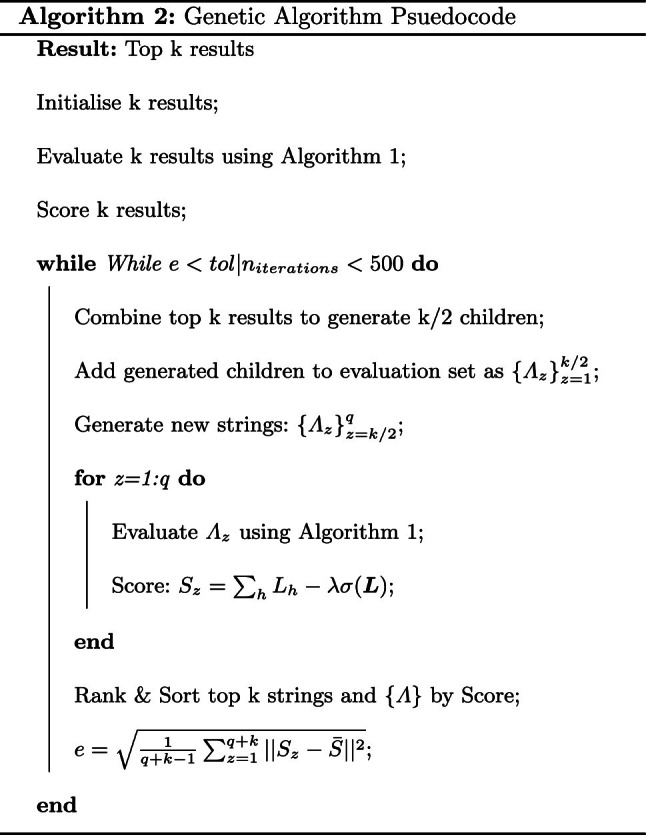


**Table 2:** Literature data on particulate emissions from talking and breathing gathered from [38]

Mode	Concentration	Size range
	N/$cm^{-3}$	$\mu$m
Breathing	0.098	0.3–20
Talking	1.41	0.3–20
Whisper	0.803	0.3–20
Coughing	0.678	0.3–20

**Fig. 1:**
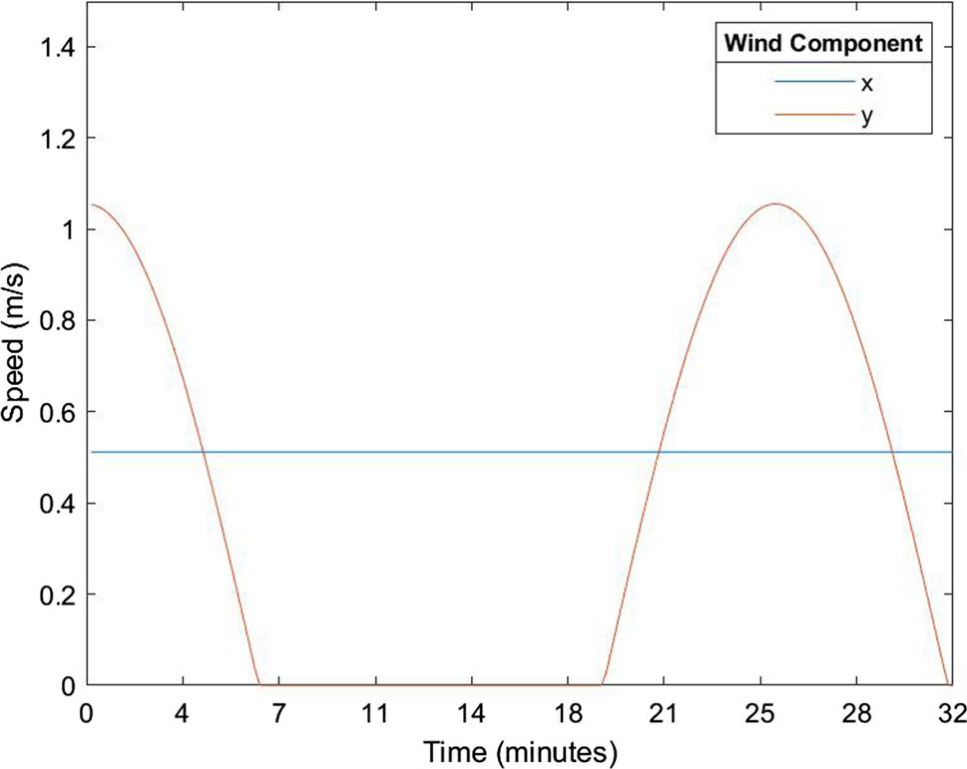
Wind velocity over time and concentration

**Fig. 2:**
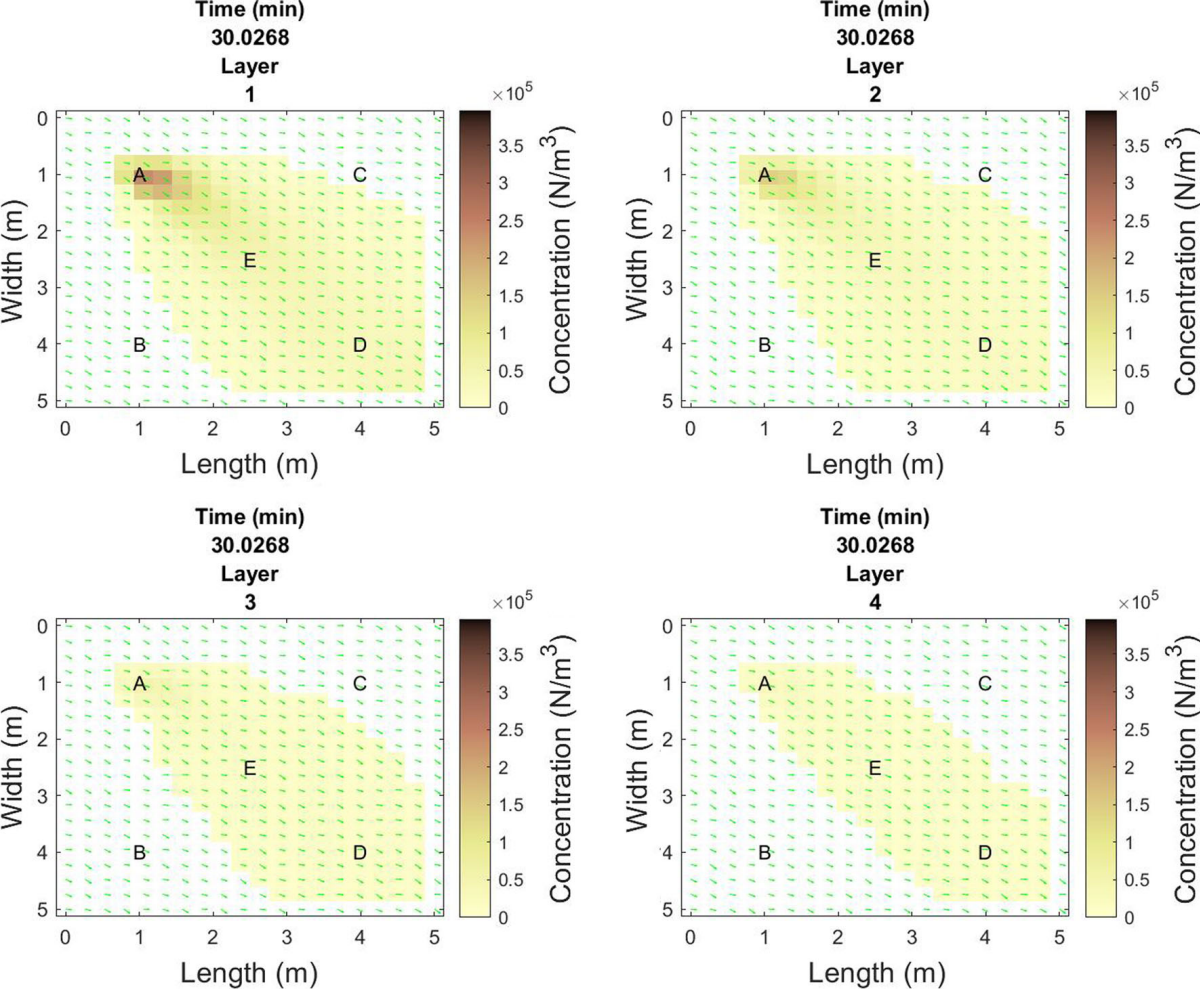
Virus particle size concentration in the space at T=30 minutes of talking by infectious person A, where layer 1-4 represent particles of diameter size [1,2,5,10]$\mu$m, respectively

**Fig. 3:**
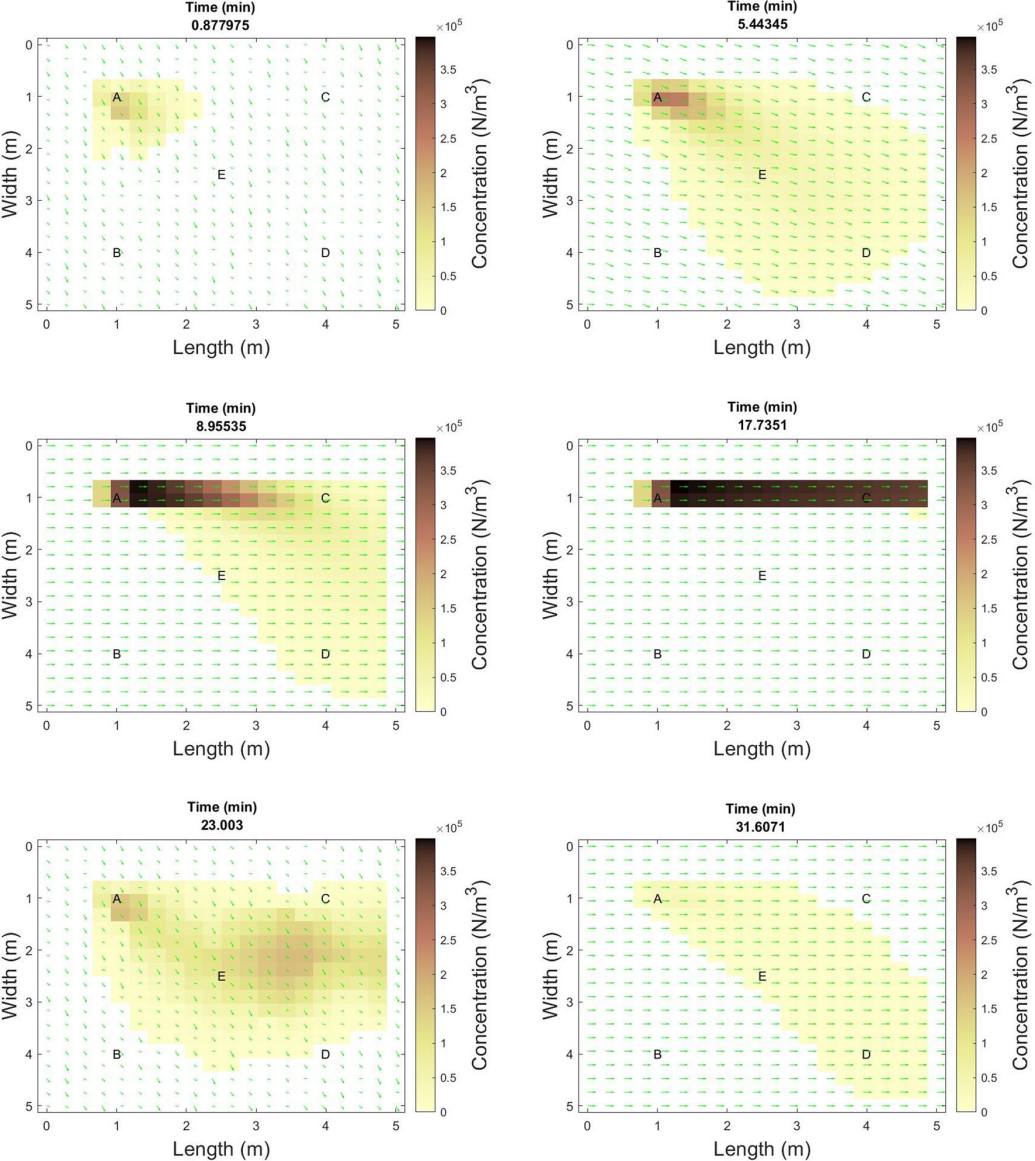
Snapshots of infectious particle distribution for particle size layer 1 ($d=1\mu m$) at .9, 5, 9, 18, 23, and 32 minutes

**Fig. 4:**
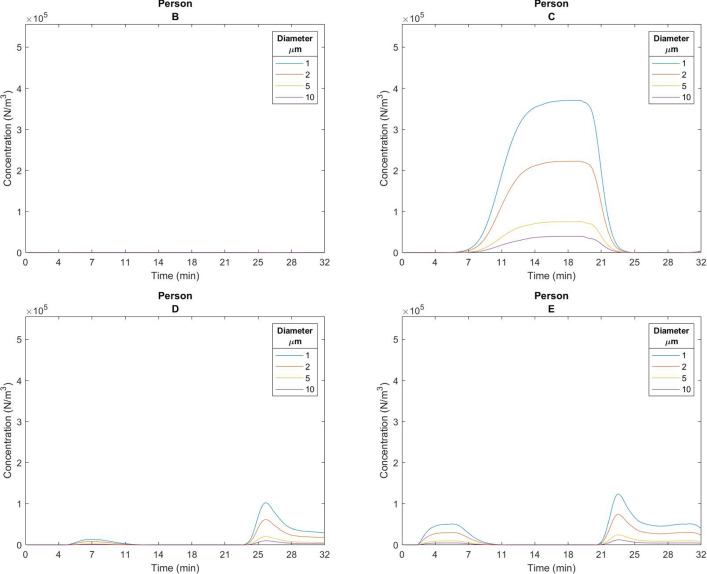
Concentration over time at each person’s location. Person B was not exposed due to the wind conditions

where ${\mathcal {F}}$ is the set of functions from Algorithm 1, $L_{h}$ is the cumulative viral load in person *h*, *N* is the number of tracked people, $\sigma$ is the standard deviation operation, $\lambda$ is the penalty factor, and $S_{z}$ is the score for string *z*. The choice of $\lambda$ is common in regularised optimisation problems and represents the weighting on divergence in the virus load values in addition to the cumulative total. A value of 0 would recreate a simple global maximising function, whereas a higher $\lambda$ would move the results toward an egalitarian result where each person is forced to have a comparable exposure total. At the limit of an infinite $\lambda$, the standard deviation would be zero and the viral load on all people would be equivalent.

For both case studies, data are extracted from the existing body of work on aerosols, exhalation, and inhalation. Particles emitted during breathing and talking indicate a range of concentration levels based on activity. Talking is used in case study results from Table 2.

## Results

### Case study 1: numerical example

An exemplar simulation is presented to demonstrate the performance of the base framework for particle generation and transport. A total of five people are spaced around a 5x5m environment. A single infected and contagious person, person A, is located approximately upwind of people B-E. A light oscillating wind condition is imposed on the environment. Person A sheds viral particles and those particles are distributed around the space. The exposure to viral particles is tracked for each of the people in the area. Input variables to this case study are:

*Input variables*

- $${\varvec{{d}}}= \lbrace 1,2,5,10\rbrace \mu m$$
- $$n = \lbrace 52,32,11,5\rbrace \%$$
- $$\rho = 1$$ g ml$$^{-1}$$
- $$T_{abs} = 273$$ K
- $${\varvec{{v}}}= (.5,1.1\cos (\omega _{y}t))$$ m s$$^{-1}$$
- $$N_{ppl} = 5$$
- $$\mu = 1.729e-5$$ kg m$$^{-1}s^{-1}$$
- $$x_{room} = 5$$ m
- $$y_{room} = 5$$ m
- $$r = 0.0128$$

The exposure window is set at 32 minutes. Dirichlet boundary conditions are used with $u=0$ at the edge of the environment. Aspirate density is set the same as water, though it can vary from .6 to 1.6 g/ml [39]. Outdoor city wind velocity is taken from San Francisco based data were maximum wind speed is taken as 2.8 m/s (10 km/h) [40]. Only low wind flow is considered as high wind conditions are likely to disperse viral particles more quickly and thus be of less concern. A small random component is added to the wind conditions to represent low-flow mixing in an outdoor environment. The wind direction is nominally in the (1,1) direction of the plot and oscillates in magnitude only in the y-direction, with a constant x component (Fig.  1).

After 30 minutes of talking, the distribution of infectious particles emitted by person A for each of the 4 layers of particle diameter size is shown in Fig.  2. The smaller distribution of larger particles is aligned with expectations due to the smaller number of large particles and how far these particles travel before sinking to the ground.

Examination of the concentration in the environment over time further highlights the strong effect of wind in carrying small particles. A series of six snapshots of viral particle concentration of the smallest particle size shows a strong following of the wind trajectory, with initial spread occurring in roughly the (1,1) direction. When the wind in the y-direction goes to zero for several minutes, the strong x-direction wind carries all particles directly from person A toward person C. As the wind shifts again, the concentration is distributed again. (Fig.  3)

Tracking exposure for each person is the key interest of the simulation and thus, a person-level examination of the concentration over time is presented (Fig.  4). This shows accurate following of the instantaneous exposure at each person’s location over time, largely due to wind carrying particles. Person B, was not exposed due to the wind preventing direct air travel from person A to person B. Person C has the highest exposure quantity and duration, as expected from the wind profile. The results show the concentration of viral particles increases over time for those downwind of the emitter. The presence of wind helps to reduce the concentration when active. Concentration build-up is dependent on the dynamics of emission rate and wind velocity. With higher wind speed, particle concentration build-up is reduced. This base model for viral particle generation dispersion and absorption is used in examining a real event next.

**Fig. 5:**
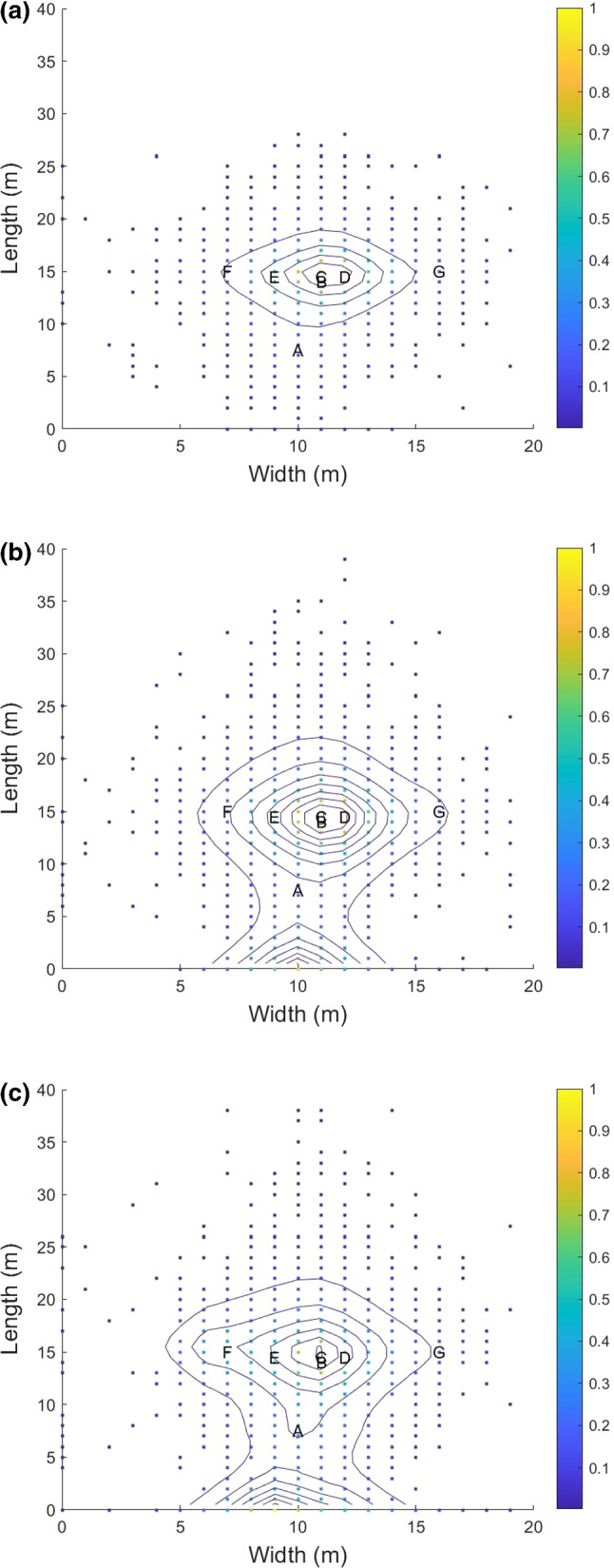
Density of likely virtual source locations for a 30,60,90 min exposure windows. (Color figure online)

### Case study 2: outdoor speaking event

The base model in Algorithm 1 is applied in an example case study to evaluate super-spreading event. The example is inspired by the outdoor speech announcing at the Rose Garden in Washington, D.C. on 25 September 2020. At this high profile event several attendees were later discovered to have contracted COVID-19 from the spreading of the SARS-CoV-2 virus: one of the main speakers and six attendees seated toward the front of the audience [37]. While spreading of this illness could have occurred elsewhere in the events of the day, this example seeks to investigate the potential for spread at such outdoor speeches. In this example, transmission occurred during the outdoor portion of such a speaking event and the likely location of a single source spreader is the desired information. A genetic algorithm is employed to compute the likely locations as described in Algorithm 2.

The locations of the 7 initial known cases are fixed in place based on their seating arrangement and speaker location. The location of a virtual 8th person is injected into the system. This virtual 8th person represents the infected and emitting individual, i.e. the super-spreader. The goal of the simulation is to find the location of this individual. Thus the location of the virtual person is allowed to freely vary throughout the entire space. Wind conditions are also allowed to vary in the search space. Video footage of the event indicates a very mild to stagnant breeze with no discernable direction [41]. Wind conditions in this case study are updated from a neutral model where occasional wind is experienced for each scenario. Wind is treated as a periodic event, thus the breeze is given an input forcing function of:

23 $$\begin{aligned} v_{x}= & {} A_{x} + B_{x} \cos (\omega _{x} t) \end{aligned}$$

24 $$\begin{aligned} v_{y}= & {} A_{y} + B_{y} \cos (\omega _{y} t) \end{aligned}$$

where *A*, *B*, and $\omega$ are constants that allow simulation of a large variety of wind conditions in the local environment. Video footage of the event suggests the wind in the speaking area is very light, thus the search space for the wind velocity is kept below 1 m/s in any direction. The algorithm is set to search for the maximum cumulative particle exposure across all particle sizes to the 7 known cases. Parameter values that differ from the simulation in Sect. 3.1 are:

**Fig. 6:**
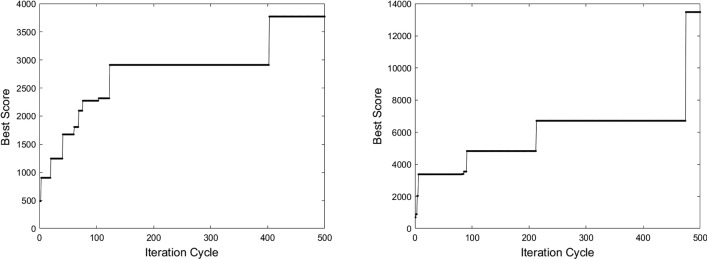
Highest scoring string after each iteration for 30 min (left) and 90 min (right) exposure windows. Each iteration represents 105 new strings evaluated

*Input variable sample ranges*

- $$A_{x} = [-.5,.5]$$ m s$$^{-1}$$
- $$B_{x} = [-.5,.5]$$ m s$$^{-1}$$
- $$A_{y} = [-.5,.5]$$ m s$$^{-1}$$
- $$B_{y} = [-.5,.5]$$ m s$$^{-1}$$
- $$\omega _{x} = [0,1 ]$$
- $$\omega _{x} = [0,1 ]$$
- $$x_{h=8} = [0,20 ]$$ m
- $$y_{h=8} = [0,40 ]$$ m

From the time-dependent exposure levels we can integrate over each particle layer and the absorption probability to compute an uptake. When an individual or universal threshold value is determined and available from future medical or other studies, it can be used in this framework to compute infectious spread. The locations of the known infections are indicated with letters A to G. The uncertain parameters in the model are the wind speed and direction, the location of the source, and the duration of exposure.

The genetic algorithm framework produces random sets of variables from the uncertain parameters and computes a score that maximises the exposure to the known locations. The highest scoring sets of parameters are kept. New guesses are produced both randomly and from the highest scoring sets. The results for 30, 60, and 90 minute exposure intervals are produced. The contours indicate the highest scoring super-spreader locations (as in Fig. 5). As the colors get warmer and contours increase, the score increases.

These results indicate if a super-spreader infected the known cases during the outdoor speech, then the spreaders would likely be located on or behind stage or near the center of the audience just behind the first two rows. The highest scoring locations for 30 and 60 minute exposure windows is the center of the audience just behind the later found infections. This matches intuition as the maximum wind velocity was limited to a low level, the ability for particles to spread to the known cases in a short to moderate time is limited. For the 60 minute exposure window a growing high scoring location is behind stage. This location represents sampled strings with a wind condition that points toward the audience. For the 90 minute exposure window, the behind stage and edge of the event space have growing high scoring results. This shows consistency with known health recommendations to reduce interaction duration as the spread of viral particles (and inhaled concentration) increases with exposure time allowing the source emitter to be further away and increase the exposure area.

The genetic algorithm is monotonic in the score improvement, indicating it is operating correctly and will converge to maximise the cost function (Fig.  6).

The results are consistent with previous work: particles tend to travel with the wind and a build-up of viral concentration is a dynamic balance of emission rate, catchment, decay of particle virality, and environmental conditions. High emitters would generate higher quantities of particle build-up, this is of particular note with the increasing awareness of super-spreaders [42, 43]. This indicates a need for a particle catchment apparatus (like a face mask) either on the emitter or protection on others to reduce inhalation. This result applies whether this model is used for infections diseases, air pollution, or noxious gasses in the range of simulated particle sizes.

This model is useful for rapid simulation of different environments and distribution of emitters and non-emitters. The simulation is rapid in computation with Case Study 2 requiring only 0.034 seconds per string for a 90 minute exposure window and 0.025 seconds for a 30 minute exposure window when implemented in MATLAB R2019a on a standard office laptop (Intel i5 1.7 GHz, 16 GB RAM). Thus, the results in this framework can be produced in minutes to narrow the search space for higher fidelity simulations for detailed particle interactions. This useful tool can be used to tune viral particle emission, absorption, and decay parameters as more data become available. Currently, these values are under-studied for SARS-CoV-2. The values used in this manuscript are from recently published works that attempt to quantify these difficult experimental quantities. For example, high fidelity and computationally expensive simulations that demonstrate the use of masks in reducing particle emission can be directly used in rapid simulation of scenarios with people in the environment with this model. The particle emission parameter for those with masks on can be updated and a new scenario computed to evaluate the dispersion of particles with the new condition as these specific quantities are better understood. This framework can also be adapted to environmental emissions or other particulate dispersions.

## Conclusions

A framework was developed to aid in the rapid simulation of viral particle spreading events. A base model that incorporates analytical and experimental data to estimate diffusion and dispersion in low-wind environments for aerosolized particles. This model is embedded into a genetic algorithm to search for a super-spreader at a spreading event. This model can be used for pollution or infectious disease estimation in rapid simulation of particle dispersion. This model can also be used to fit results to new and developing data on infectious particles or to rapidly simulate different scenarios using existing data. Computational cost of new arrangements scales with wind velocity, environment size, and the number of bins in the discretization of particle sizes, but not with the number of particles. This de-coupling from the individual particles provides a substantial reduction in the computation time and great flexibility in utilization.

## Future work

Additional data on target particle properties in air and emission/absorption parameters is needed for more accurate estimation. Additional data on super-spreaders and particle decay rate are needed for more refined estimates.

## Data Availability

Code is not publicly available.

## List of symbols

$\alpha$: Portion of particles assigned to each size bin
$\varvec{\nu }$: Effective particle velocity
$\dot{b}$: Breathing rate (breaths/min)
$\gamma$: Viral density in exhaled particles (#/mL)
$$\mu $$: Fluid viscosity
$$\rho $$: Density
*A*: Area
*a*: Absorption coefficent
$C_{S}$: Cunningham slip correction factor
$$d_{p}$$: Particle diameter
*h*: Index for each person
*K*: Particle sink
$$k_{Boltz}$$: Boltzmann constant
*L*: Total viral load in a person
*m*: Number of particle size layers/bins
*N*: Number of tracked people
*R*: Particle source
*r*: Viral decay rate in the environment by size layer
*s*: Aspirate expulsion rate (mL/breath)
$$T_{abs}$$: Absolute temperature
*u*: Infectious particle concentration
*x*, *y*: Cartesian coordinates locations
D: Diffusion constant
i,j,l: Indices for each spatial location and particulate size layer
